# Supplementary material for: Relationship between physical activity and neighborhood environment in preschool children during COVID-19.—A cross-sectional study using 24-hour activity records-
Source: PLoS One. 2024 Oct 29;19(10):e0304848. doi: 10.1371/journal.pone.0304848 (PMC11521261; doi:10.1371/journal.pone.0304848)
Supplement: Attachment — Submitted filename: rebuttal_letter.doc [file pone.0304848.s001.doc]

[July 25,2023]

Emily Chenette

Editor-in-Chief

*PLOS ONE*

Dear Editor:

Thank you for inviting us to submit a revised draft of our manuscript entitled, “[Relationship between physical activity and neighborhood environment in preschool children during COVID-19.

- A cross-sectional study using 24-hour activity records-]” to [PLOS ONE]. We also appreciate the time and effort you and each of the reviewers have dedicated to providing insightful feedback on ways to strengthen our paper. Thus, it is with great pleasure that we resubmit our article for further consideration. We have incorporated changes that reflect the detailed suggestions you have graciously provided. We also hope that our edits and the responses we provide below satisfactorily address all the issues and concerns you and the reviewers have noted.

To facilitate your review of our revisions, the following is a point-by-point response to the questions and comments delivered in your letter dated July 25, 2023.

**Review Comments to the Author**

I would like to express my heartfelt gratitude to the reviewers for their meticulous evaluation of the manuscript. Their constructive and thoughtful comments have been immensely valuable in identifying areas that would benefit from improvement and providing insightful suggestions to enhance the overall quality of the paper. The feedback received has undoubtedly contributed to strengthening the scientific rigor and relevance of the study.

1.Please use the space provided to explain your answers to the questions above. You may also include additional comments for the author, including concerns about dual publication, research ethics, or publication ethics. (Please upload your review as an attachment if it exceeds 20,000 characters)

Reviewer #1:

**RESPONSE**： Thank you for dedicating your time and effort to reviewing my paper. Your feedback was incredibly helpful in improving the manuscript. I appreciate your thoughtful and constructive comments, which strengthened the research.

Abstrat

The first paragraph of the results section should be in the methods section.

**RESPONSE：** Thank you for this suggestion**.** We have incorporated this input and inserted the documentation of the first paragraph of the results in the "Methods" section (p. #2, lines #33-#36.).

Introduction

The introduction lacks fluidity, it’s telegraphic. It does not present the context of COVID in Japan. What do we know about COVID in Japan? What kind of measures were implemented in your country? How did these measures affected the Japanese children and their routines?

**RESPONSE：** Thank you for this suggestion**.** We have incorporated these comments, presented the context of COVID-19 in Japan, and reorganized all introductions (p. #2-#4, lines #47-#88.).

Methods

Instrumentation

A questionnaire sample should be presented for replication of the study.

**RESPONSE：** Thank you for this suggestion**.** The questionnaire sample is shown in the attached　table with the results.

Correlation Between Physical Activity Time and Questionnaire

The authors found correlations between some questions and SET, SCT, IP, OP and Go. But which one of the variables was the best predictors of IP, OP, GO and SET?

It would be interesting to know this.

**RESPONSE：** Thank you for this suggestion**.** In the questionnaire, "screen time: has your current screen time changed compared to that in January 2020 (before the COVID-19 pandemic)," was the best factor in the study, correlating with SET, SCT, and OP (p. #8-#9, lines #185-#209.).

Results

The third paragraph should be in the discussion section

**RESPONSE：** Thank you for this suggestion. The sentence in the third paragraph was deleted and replaced with a sentence containing only the R2 value and prediction accuracy results. Therefore, we did not let it be inserted in the discussion section, but inserted it in the results section. (p. #10, lines #228-#229.).

Discussion

In the “About Physical Activity Time”, the results are not discussed. The authors limit their text to point out the harmful health effects of the numbers founded but, they don’t discuss the “whys” of it. The reader needs to understand why these numbers appear in Japan. What reasons do the authors believe led to the results founded.

**RESPONSE：** Thank you for this suggestion**.** We have reflected this comment by Children's PA time is usually ensured by outdoor activities, but the effect of COVID-19 restrictions may have reduced PA time and increased SCT (p. #10-#11, lines #237-#248).

2.In the second paragraph of “From the results of multiple regression analysis” section, the authors affirm “Many parents refrained from going out, and thus used handheld gaming consoles and tablets to keep their infants occupied.”, can you prove this? Is this a citation?

Again, the authors do not discuss the “whys” …

**RESPONSE：** Thank you for this suggestion**.** We have incorporated these comments, showing previous studies on the relationship between PA and the neighborhood environment, and restructured all discussions. (p. #10-#12, lines #232-#273).

Reviewer #2: The data used in this research were limited to a certain holiday, and within a certain time period, which might cause some bias in interpreting the results. Also, the stepwise regression might not be an appropriate analytical instrument for this research. However, this research did show an acceptable result aside from the above limitations.

**RESPONSE：** Thank you for this suggestion**.** We agree with this comment and have added it to the limitation sections (p. #12-#13, lines #288-#291).

Reviewer #3:

**RESPONSE：** I have separated my responses to the reviewers’ comments according to several categories in order to achieve an integrated approach in my responses.

1. The tables were not included with the submission, and thus it is not possible to assess the data and results of the study.

**RESPONSE：** Thank you for this suggestion**.** We have included a table in the submission.

2. In the text, the terms "infants, toddlers, and children" are not consistent, and the age range of the participants is unclear.

**RESPONSE：** Thank you for this suggestion**.** We have incorporated this comment and unified the term "infants, toddlers, and children" with the term "preschoolers.

3. The meaning of "environment" in the text is unclear.

**RESPONSE：** Thank you for this suggestion**.** In this paper, the meaning of "environment" is in the sense of Physical location

4. There is a topic(for example, parental lifestyle) in the conclusion of the abstract that is not included in the text.

**RESPONSE：** Thank you for this suggestion**.** We have reflected this comment by removing sentences from the abstract that are not addressed in the text, such as parental lifestyle, and replacing them with sentences that are addressed in the text (p. #2, lines #40-#42).

5. The introduction sections that "the number of physically inactive children has been on the rise," but the rationale should be provided.

**RESPONSE：** Thank you for this suggestion. We will reflect these comments in our efforts to promote physical activity among children in Japan (p. #3, lines #62-#72).

6. It does not state what "environment" means.

**RESPONSE：** Thank you for this suggestion**.** Please see point 3 above.

7. It is unclear when "holiday" means.

**RESPONSE：** Thank you for this suggestion**.** In this paper, holidays are defined as sunny days without special events such as Saturday or Sunday trips (p. #5-#6, lines #111, #121-#122).

8. There is a lack of information of 24hPAR.

**RESPONSE：** Thank you for this suggestion. We have reflected on this comment and used prior research to illustrate the 24hPAR (p. #5, line#107-#109, #117-#126).

9. Some categories show % but others do not. There should be consistency.

**RESPONSE：** Thank you for this suggestion. We have reflected this comment and removed all % notations from the results.

10. The questionnaire says "compared to January 2020 (before the COVID-19 outbreak)," but I don't know what this refers to.

**RESPONSE：** Thank you for this suggestion**.** For example, "screen time, compared to January 2020 (before the COVID-19 outbreak)" was changed to "has your current screen time changed compared to that in January 2020 (before the COVID-19 pandemic)." Hope this clarifies the unclear.

11. The manuscript should be edited for grammar and sentence structure.

**RESPONSE：** Thank you for this suggestion. We agree with you and have incorporated this suggestion throughout our paper. We have reorganized most of this paper and requested a third-party (editage) review for improvement.

Again, thank you for giving us the opportunity to strengthen our manuscript with your valuable comments and queries. We have worked hard to incorporate your feedback and hope that these revisions persuade you to accept our submission.

Sincerely,

[Author’s name]　NAOKI YAMAGUCHI

[Affiliation] Grandplaza itibankan 949 konono Hashimoto city Wakayama prefecture

[Postal address]648-0086

[Phone number] +8190-5135-1325

[Email address] f0985561@kio.ac.jp
